# A model integrating *CEA* and *KRAS* mutation statuses and computed tomography tumor-habitat radiomics predicts synchronous liver metastases in patients with rectal cancer: a dual-center study

**DOI:** 10.3389/fonc.2026.1927131

**Published:** 2026-10-28

**Authors:** Yunhua Shi, Jinge Li, Deqiu Ye, Weiqiang Shen, Mingfang Hu, Jian Shen, Guanfeng Chen

**Affiliations:** 1 Radiology Department of Huzhou Central Hospital, Fifth School of Clinical Medicine of Zhejiang Chinese Medical University, Huzhou, China; 2 School of Medicine & Nursing, Huzhou Normal University, Huzhou, China; 3 Radiology Department of Quanzhou First Hospital Affiliated to Fujian Medical University, Quanzhou, China

**Keywords:** computed tomography, habitat analysis, radiomics, rectal cancer, synchronous liver metastasis

## Abstract

**Objective:**

To investigate the diagnostic performance of a computed tomography (CT)-based habitat radiomics model for discriminating between patients with rectal cancer (RC) with and without synchronous liver metastases (SLM).

**Methods:**

This retrospective study enrolled two independent patient cohorts. Cohort 1 (internal cohort) included 198 patients with pathologically confirmed RC with complete CT data from April 2022 to June 2025, comprising 46 SLM-positive and 152 SLM-negative cases. This cohort was divided randomly in a 7:3 ratio into a training set (140 patients: 33 SLM-positive, 107 SLM-negative) and an internal validation set (58 patients: 13 SLM-positive, 45 SLM-negative). Cohort 2 served as an external validation set, consisting of 55 patients with RC (18 SLM-positive, 37 SLM-negative). Regions of interest (ROIs) were delineated manually slice-by-slice on arterial-phase thin-slice CT images. Raw radiomics features were extracted from the ROIs and K-means clustering was implemented to segment tumor sub-habitats, followed by feature extraction for each sub-habitat. Feature-dimensionality reduction was performed on the training set using minimum redundancy maximum relevance and least absolute shrinkage and selection operator algorithms. Logistic regression classifiers were constructed to develop a conventional radiomics model and a habitat radiomics model, respectively. Model performance and clinical utility were quantified using the area under the receiver operating characteristic curve (AUC), calibration curves, and decision curve analysis (DCA).

**Results:**

Univariate and multivariate logistic regression identified serum *CEA* and *KRAS* mutations as independent risk factors for SLM. These were integrated to build a clinical baseline model with AUC values of 0.730 (training set), 0.724 (internal validation set), and 0.701 (external validation set). After rigorous feature screening, 13 conventional radiomics features and 13 habitat radiomics features were retained for model construction. The conventional radiomics model yielded AUCs of 0.785 (training), 0.764 (internal validation), and 0.782 (external validation), while the habitat radiomics model achieved higher AUCs, with AUCs of 0.862, 0.826, and 0.826, respectively. The combined model integrating independent clinical risk factors and habitat radiomics score attained the numerically highest AUCs, with AUCs of 0.883 (training), 0.846 (internal validation), and 0.851 (external validation), outperforming the clinical, conventional radiomics, and standalone habitat models with robust cross-cohort stability. Calibration curves demonstrated favorable predictive consistency of the combined model, while DCA revealed sustained clinical net benefit across broad risk-threshold ranges.

**Conclusion:**

A CT-based habitat radiomics model could effectively identify patients with RC with SLM, with performance numerically better than that of conventional whole-tumor radiomics. Integrating clinical biomarkers with habitat radiomics further increased the predictive accuracy, offering a novel non-invasive preoperative risk-stratification strategy for RC.

## 1 Introduction

Rectal cancer (RC) is a prevalent gastrointestinal malignancy with substantial global morbidity and mortality, posing a severe burden to human health (1, 2). Distant metastases of RC predominantly occur in the liver, with synchronous liver metastases (SLM) detected in 15%–20% of newly diagnosed patients at initial presentation and constituting a leading cause of disease-related mortality (3, 4). The median overall survival of patients with untreated colorectal liver metastases in the absence of standardized multimodal therapy is only 5–20 months (5). The timely and accurate identification of SLM at baseline thus enables clinicians to formulate individualized therapeutic regimens and improve long-term patient survival (6, 7).

Contrast-enhanced computed tomography (CT) is a routine preoperative imaging modality for RC, providing comprehensive morphological, densitometric, and contrast-enhancement information for primary tumors. Radiomics is an evolving technology that allows the high-throughput extraction and analysis of imaging features associated with tumor heterogeneity, facilitating the investigation of correlations between these features and relevant biomarkers (8–10). Artificial intelligence-driven radiomics enables the high-throughput quantification of tumor phenotypes that are invisible to human radiologists, exhibiting promising translational value for tumor subtyping and prognostic stratification (11–14). Conventional radiomics extracts quantitative signatures from the entire tumor bulk, thus inherently ignoring intratumoral spatial heterogeneity. Tumor-habitat analysis has emerged as an advanced analytical framework that partitions regions of interest (ROIs) into distinct subregions via unsupervised clustering of voxels with homogeneous gray-scale intensity. This methodology captures spatially resolved intratumoral heterogeneity, and can thus help to elucidate the biological mechanisms underlying tumor invasion, metastasis, and therapeutic resistance (15–18).

This study aimed to explore the identification of SLM in patients with RC based on a CT-based habitat analysis model, to evaluate the efficacy of the model, and provide clinical evidence for early intervention and comprehensive diagnosis and treatment decision-making.

## 2 Materials and methods

### 2.1 Patient cohorts

This retrospective, dual-center study enrolled consecutive patients with pathologically confirmed RC at two independent institutions. The primary cohort (Center 1) was sourced from Quanzhou First Hospital Affiliated to Fujian Medical University, and included patients with clinical and CT data collected between April 2022 and June 2025. SLM status was verified by postoperative histopathology or contrast-enhanced liver magnetic resonance imaging (MRI). The external validation cohort (Center 2) comprised patients from Huzhou Central Hospital, Fifth School of Clinical Medicine of Zhejiang Chinese Medical University, who were enrolled from May 2023 to December 2025.

The inclusion criteria were histopathological confirmation of primary rectal adenocarcinoma with definitive SLM status confirmed by pathology or MRI, Received unenhanced and dual-phase enhanced CT of the whole abdomen within 2 weeks before surgery, and complete preoperative abdominal CT imaging with satisfactory image quality. Patients with neoadjuvant chemotherapy or radiotherapy administered prior to CT acquisition or with concurrent or prior history of other malignant neoplasms were excluded.

After screening according to the inclusion and exclusion criteria, a total of 198 patients were finally enrolled at Center 1 (**Figure 1**). There were 128 males and 70 females (age 28–86 years), including 46 patients with and 152 patients without SLM. A total of 55 patients from Center 2 were included as the external validation group, including 37 males and 18 females (age 48–84 years), of whom 18 had SLM and 37 had no SLM. This retrospective study was approved by the Ethics Committees of Quanzhou First Hospital Affiliated to Fujian Medical University (Approval No.: Quan-Yi-Lun-2024-K133) and Huzhou Central Hospital, Fifth School of Clinical Medicine of Zhejiang Chinese Medical University (Approval No.: 2026-124(Research)-01). The study complied with the requirements of the Declaration of Helsinki, and informed consent from patients was waived. To reduce the risk of missing or non-uniformly recorded clinical data arising from the relatively long case-collection period, we deliberately selected a limited set of core clinical indicators with complete medical records. All patients enrolled in the final cohort had no missing values for these selected clinical and imaging variables.

**Figure 1:**
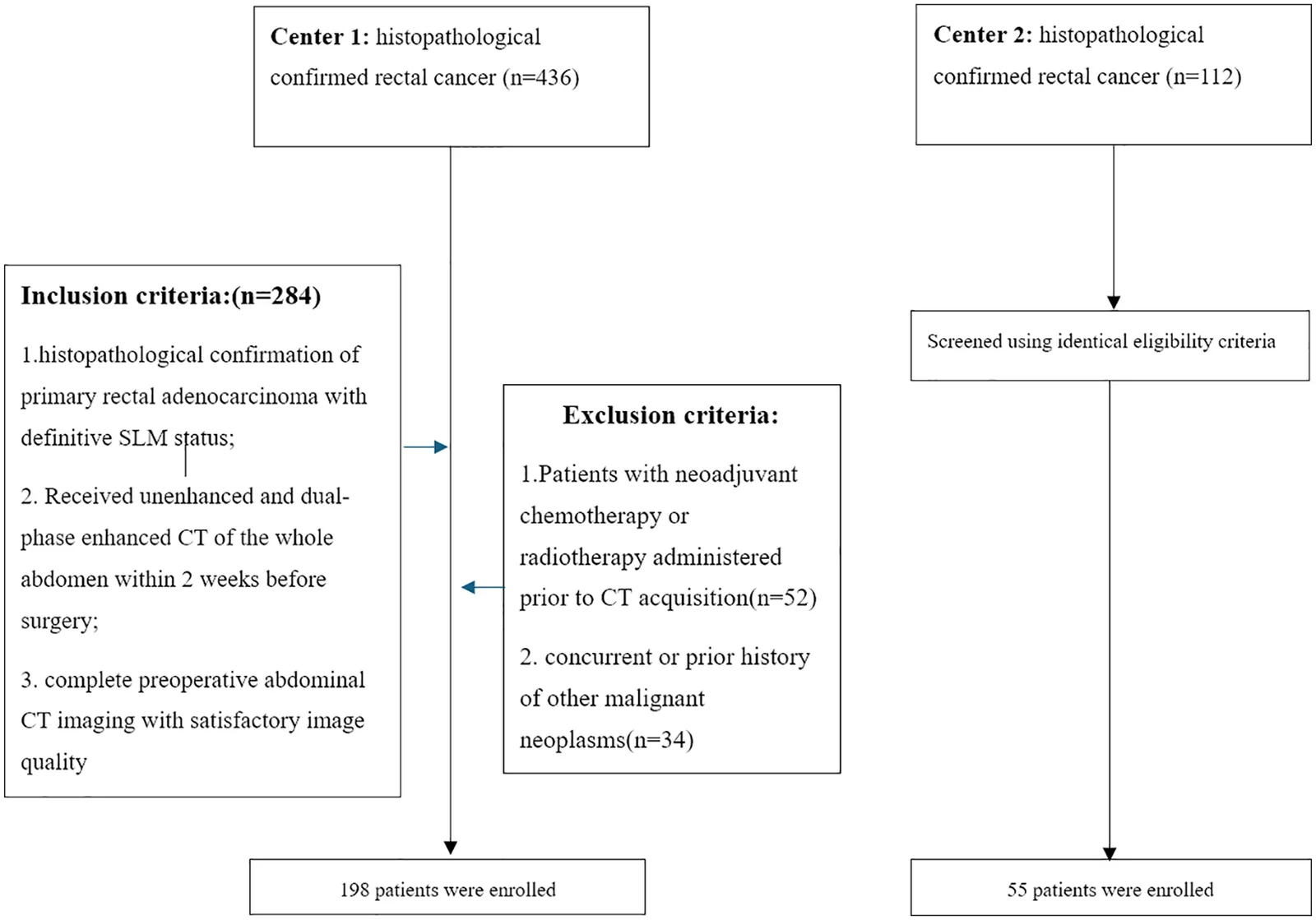
Flowchart of inclusion and exclusion of research subjects.

### 2.2 Instruments and scanning protocols

Routine whole-abdominal scanning was carried out using a GE 64-slice CT scanner (Optima CT660, General Electric, USA). Patients were placed in a supine position, with a scanning range from the dome of the diaphragm to the lower edge of the pubic symphysis. The scanning parameters were as follows: tube voltage 120 kV, automatic tube current, slice thickness 5 mm, and slice interval 5 mm. Enhanced scanning was carried out using the time method: after plain scanning, iohexol (iodine concentration: 300 mg/mL) was injected via an antecubital vein cannula using a high-pressure injector at a flow rate of 3.0 mL/s. Arterial- and venous-phase scans were initiated at 30 s and 60 s after the start of injection, respectively.

The external validation cohort from Center 2 was scanned on a GE Light-speed VCT 64-slice 128-channel spiral CT scanner (GE, United States). The abdominal routine scan protocol was 120 KV tube voltage, 200 mA tube current, with a slice thickness of 5.0 mm and slice interval of 5.0 mm.

KRAS mutation analysis was performed using the amplification refractory mutation system polymerase chain reaction (ARMS‐PCR). The assay covered exons 2, 3 and 4 of the KRAS gene. A sample was defined as KRAS‐mutant if a positive signal for any target mutation was detected according to the manufacturer’s instructions.

### 2.3 Delineation of ROIs

The voxel spacing of arterial-phase CT images for all patients was resampled to 1 mm × 1 mm × 1 mm to achieve uniform spatial resolution. ComBat harmonization was further applied to mitigate batch effects arising from subtle variations in image acquisition and reconstruction parameters across the two centers. The ROIs of the lesions were delineated by Physician A, with > 5 years of diagnostic experience, and Physician B, with > 15 years of diagnostic experience using ITK-SNAP software (version 4.2.2). The scope of tumors on the axial plane was delineated slice by slice to form ROIs. Two weeks after the initial tumor segmentation, 30 patients were randomly selected and the tumor ROIs were re-delineated independently by two radiologists. The intra-class correlation coefficient (ICC) was calculated for intra-reader reproducibility by comparing the two segmentations performed by Physician A, and for inter-reader reproducibility by comparing the segmentations of Physician A and Physician B.

### 2.4 Tumor-habitat subregion partitioning and radiomics-feature extraction

Quantitative radiomic features were extracted from segmented ROIs using the open-source Python package PyRadiomics v3.0.1. The arterial-phase CT images were resampled to a uniform voxel spacing of 1 mm × 1 mm × 1 mm using B-spline interpolation (sitkBSpline). After resampling, 3D wavelet decomposition and Laplacian-of-Gaussian (LoG) filtering (σ = 1.0 mm, 3mm) were applied. Gray-level discretization adopted a fixed bin width strategy with bin width = 25 HU. The extracted feature categories included 198 first-order histogram features, 14 morphological features, 176 gray-level run-length matrix features, 154 gray-level dependence matrix features, 242 gray-level co-occurrence matrix features, and 176 gray-level size zone matrix features, yielding a total of 960 raw radiomics features per tumor ROI. Habitat segmentation was achieved using unsupervised K-means clustering implemented via Scikit-Learn v1.6. After splitting the dataset into training, validation and external cohorts, all tumor voxels from the training cohort were pooled to train the K-means model. A range of cluster numbers (K = 2-10) was tested, and the optimal K was determined by maximizing the silhouette coefficient calculated solely on training-set voxels. The trained cluster centroids were then fixed and applied to assign habitat labels to voxels in the validation and external cohorts without refitting or re-selecting K. After partitioning each tumor into distinct habitat subregions, PyRadiomics was reapplied to extract radiomics signatures from each individual sub-habitat.

### 2.5 Feature-dimension reduction, feature selection, and diagnostic model construction

The dataset was randomly partitioned into a training set and validation set using a ratio of 7:3. Stratified random sampling was employed with tumor mutation status as the stratification criterion to ensure that the distribution of mutation rates was consistent in the training and validation cohorts. Z-Score standardization was applied to the extracted radiomics features to ensure consistency across the entire dataset. Feature dimensionality reduction was performed on the training set using a two-step approach. First, the minimum redundancy maximum relevance (mRMR) algorithm preliminarily selected the top 15 features. These were then subjected to least absolute shrinkage and selection operator (LASSO) penalized logistic regression for further feature selection and coefficient shrinkage; the penalty parameter (λ) was tuned via 10-fold cross-validation, and the optimal λ was selected using the λ.min rule. Sample size was calculated for an expected area under the receiver operating characteristic curve (AUC) value of 0.80 (**Supplementary File 1**). Diagnostic models were developed using a logistic regression classifier and the performances of different diagnostic models was assessed according to the area under the receiver operating characteristic curve. Decision curve analysis curves were plotted to evaluate the clinical net benefit of the models, model goodness-of-fit was assessed using calibration curves, and the consistency of the calibration curves were verified using the Hosmer–Lemeshow test (**Figure 2**).

**Figure 2:**
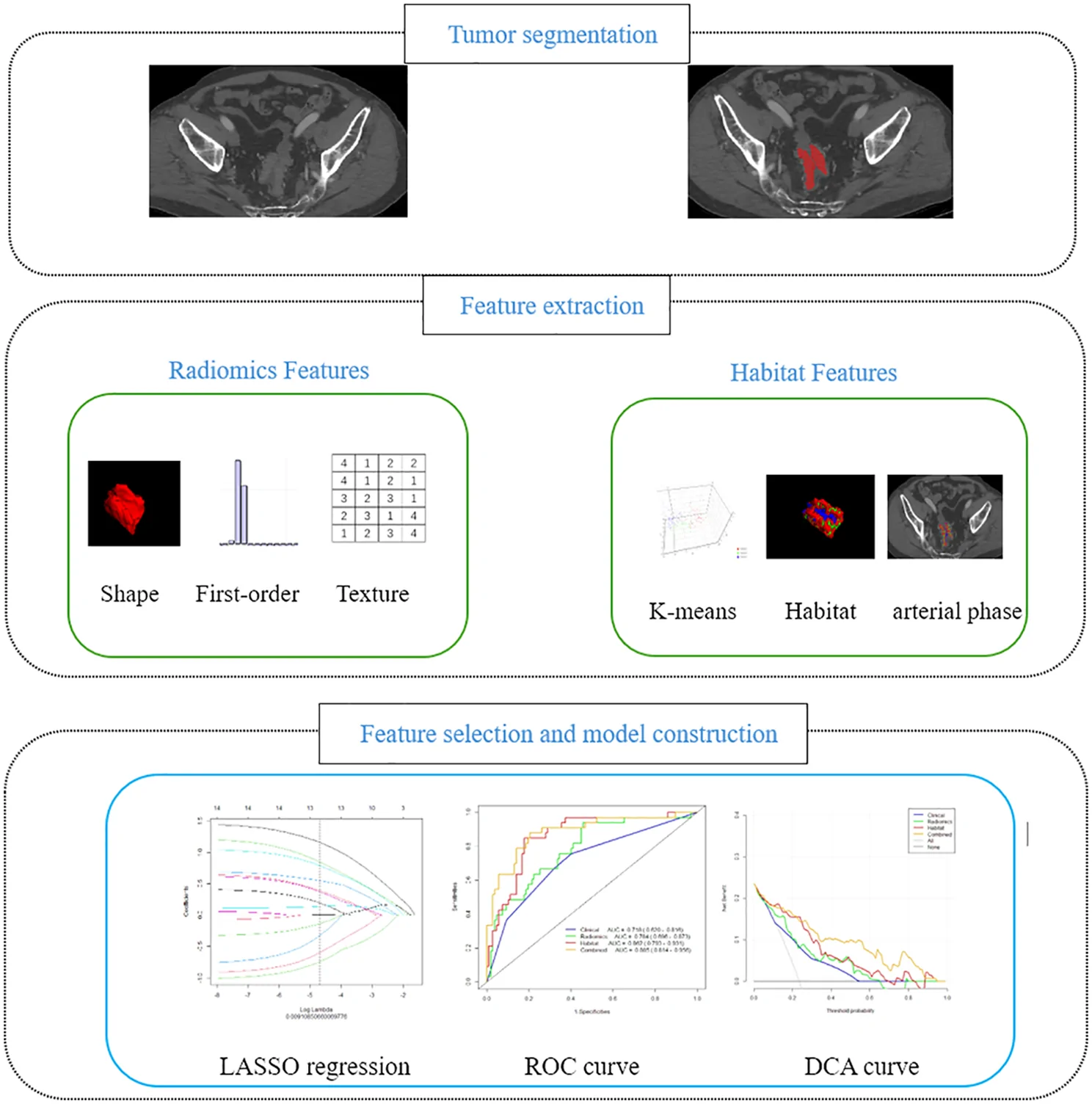
Workflow of this study.

### 2.6 Statistical analysis

Statistical analyses were performed using Python 3.12, SPSS 26.0, and R 4.2.1 software. The Kolmogorov–Smirnov test was used to assess the normality of continuous data. Normally distributed data were expressed as mean ± standard deviation, while skewed data were presented as quartiles. Categorical data were compared between groups using Pearson’s χ^2^ test, and continuous data were compared using an independent samples t-test (for normally distributed data) or Mann–Whitney U test (for skewed distributed data). P value < 0.05 was considered statistically significant.

## 3 Results

### 3.1 Baseline clinical characteristics and construction of the clinical predictive model

Center 1: A total of 198 patients with RC were enrolled in the final study, including 46 patients with synchronous liver metastasis and 152 patients without synchronous liver metastasis; In the training cohort (n=140), SLM status was confirmed by postoperative histopathology in 95 patients (67.9%) and by contrast-enhanced liver MRI in 45 (32.1%); In the internal validation cohort (n=58), the corresponding numbers were 36 (62.1%) by histopathology and 22 (37.9%) by MRI; Center 2: A total of 55 patients were enrolled in the study, including 18 patients with synchronous liver metastasis and 37 patients without synchronous liver metastasis. In the external validation cohort (n=55), 33 (60.0%) were confirmed by histopathology and 22 (40.0%) by MRI. The comparison results between the training group and the validation group showed no statistically significant differences in all indicators (P>0.05), indicating satisfactory random grouping (**Table 1**). In the training group, univariate Logistic regression analysis was performed to screen differential factors between patients with and without synchronous liver metastasis of rectal cancer. The results revealed no statistically significant intergroup differences in age, gender and CA125 (P>0.05), while statistically significant differences were observed in CEA, CA199 and KRAS (P<0.05). The clinical indicators CEA, CA199 and KRAS were incorporated into multivariate Logistic regression analysis, which demonstrated statistically significant differences in CEA and KRAS (P<0.05) but no statistically significant difference in CA199 (**Table 2**). Accordingly, a clinical model was constructed based on the clinical indicators CEA and KRAS. The AUC of this clinical model was 0.730 (0.632-0.827) in the training group, 0.724 (0.576-0.872) in the validation group, and 0.701 (0.566-0.835) in the external validation group.

**Table 1:** Comparison of clinical data between the training group and the validation group.

Factor	Training (n=140)	Validation (n=58)	Statistic	P -value	External validation (n=55)
Age, y(M ± SD)	64.04 ± 11.23	63.02 ± 11.33	0.579	0.563^t^	67.60 ± 8.94
Gender, n (Male/Female)	89/51	39/19	0.242	0.623^a^	37/18
CEA elevation, n(Yes/No)	56/84	32/26	3.823	0.051^a^	24/31
CA125 elevation, n(Yes/No)	28/112	15/45	0.829	0.363^a^	35/20
CA199 elevation, n(Yes/No)	34/106	13/45	0.079	0.778^a^	19/36
KRAS, n (mutant and wild-type)	46/94	26/32	2.539	0.111^a^	32/23
liver metastases, n (Yes/No)	33/107	13/45	0.031	0.861^a^	18/37

^t^ t-value; ^a^Chi-square test. CEA, Carcinoembryonic Antigen; CA125, Carbohydrate Antigen 125; CA199, Carbohydrate Antigen 199; KRAS, Kirsten rats arcomaviral oncogene homolog.

**Table 2:** Univariate and multivariate logistic regression analyses between groups with and without liver metastasis in the training cohort.

Factor	Univariate logistic regression OR (95%CI)	P	Multivariate logistic regression OR (95%CI)	P
Age	1.017 (0.981-1.054)	0.367		
Gender	1.429 (0.618-3.305)	0.404		
CEA	4.294 (1.872-9.851)	<0.001	3.186 (1.313-7.734)	0.010
CA125	2.150 (0.875-5.281)	0.095		
CA199	3.860 (1.657-8.992)	0.002	2.442 (0.963-6.196)	0.060
KRAS	3.386 (1.507-7.607)	0.003	2.724 (1.144-6.484)	0.024

CEA, Carcinoembryonic Antigen; CA125, Carbohydrate Antigen 125; CA199, Carbohydrate Antigen 199; KRAS, Kirsten rats arcomaviral oncogene homolog.

### 3.2 Feature selection and model construction of radiomics model

A total of 960 radiomics features were extracted from the arterial phase images of each patient. The intra-reader ICC of the 960 radiomic features ranged from 0.830 to 0.850, and the inter-reader ICC ranged from 0.820 to 0.835, dimension reduction and screening, 13 radiomics features were finally retained for radiomics model construction. The constructed radiomics model achieved an AUC of 0.785 (0.696-0.873) in the training cohort, an AUC of 0.764 (0.630-0.899) in the validation cohort, and an AUC of 0.782 (0.655-0.909) in the external validation cohort (**Figures 3**, **4**; **Table 3**).

**Figure 3:**
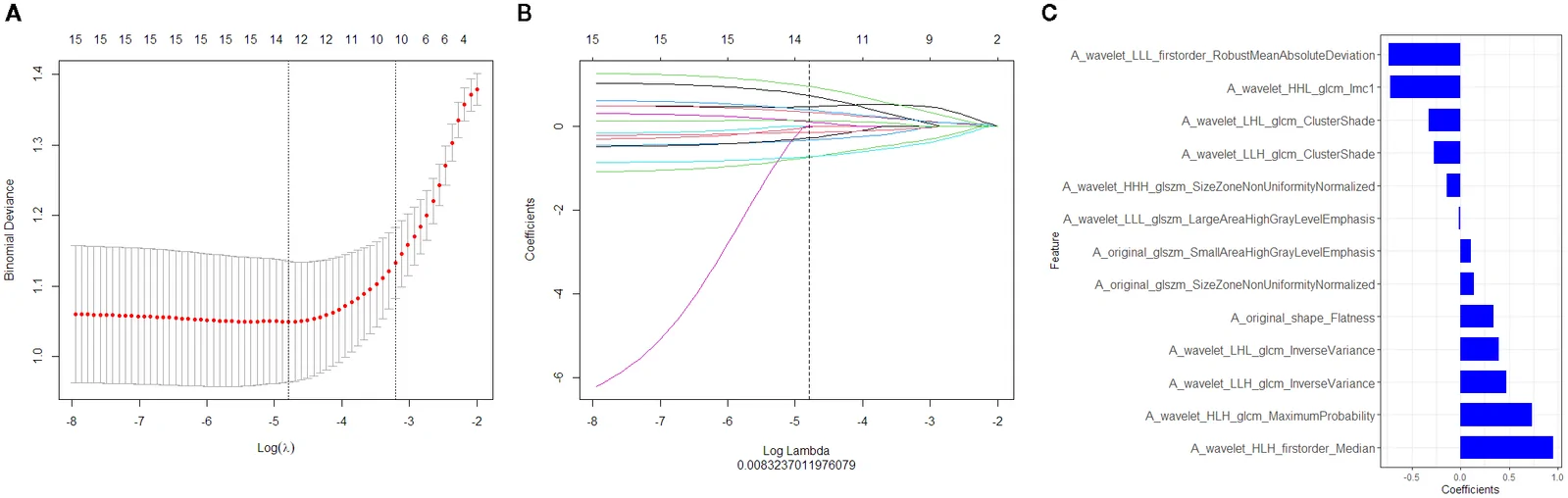
Feature dimensionality reduction and selection for radiomic models. **(A, B)** Schematic diagrams of feature selection and dimensionality reduction; **(C)** Selected features and their weights after dimensionality reduction and selection).

**Figure 4:**
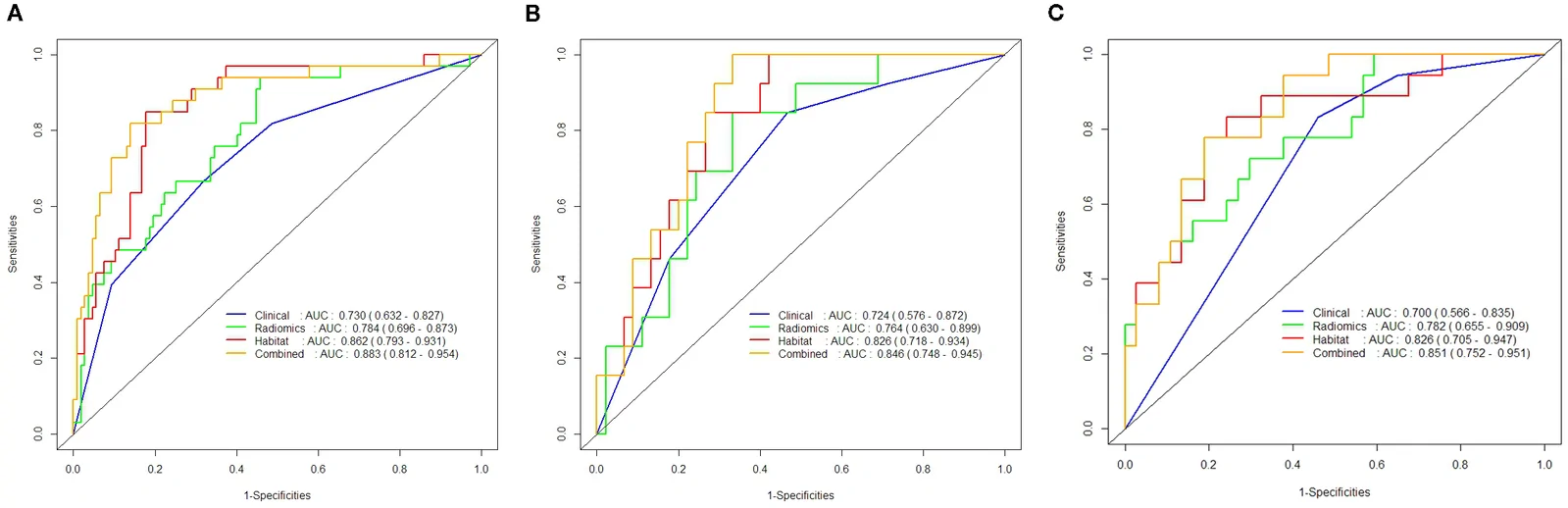
ROC curve results of radiomic diagnostic models. **(A)** Training set; **(B)** validation set; **(C)** external validation set).

**Table 3:** Diagnostic performance of radiomic models.

Model	Cohort	AUC (95% CI)	Accuracy	Sensitivity	Specificity
Clinical	Training	0.730 (0.632-0.827)	0.679	0.667	0.682
	Validation	0.724 (0.576-0.872)	0.603	0.846	0.533
	external validation	0.701 (0.566-0.835)	0.636	0.833	0.541
Radiomics	Training	0.785 (0.696-0.873)	0.636	0.939	0.542
	Validation	0.764 (0.630-0.899)	0.707	0.846	0.667
	external validation	0.782 (0.655-0.909)	0.709	0.722	0.703
Habitat	Training	0.862 (0.793-0.931)	0.829	0.848	0.822
	Validation	0.826 (0.718-0.934)	0.759	0.846	0.733
	external validation	0.826 (0.705-0.947)	0.782	0.833	0.757
Combined	Training	0.883 (0.812-0.954)	0.850	0.818	0.860
	Validation	0.846 (0.748-0.945)	0.741	1.000	0.667
	external validation	0.851 (0.752-0.951)	0.800	0.778	0.811

### 3.3 Feature screening and model construction of habitat model

In this study, the silhouette coefficients for clustering numbers ranging from 2 to 10 were calculated. The results showed that the silhouette coefficient reached its maximum value when the clustering number was 3. Therefore, the ROI was divided into three subregions in this study, and 960 radiomic features were extracted from each subregion, with a total of 2880 radiomic features. After dimensionality reduction and screening, a total of 13 features were incorporated into the final model, including 5 from subregion 1, 2 from subregion 2, and 6 from subregion 3 (h1: subregion 1, h2: subregion 2, h3: subregion 3). The habitat model constructed based on these features achieved an AUC of 0.862 (0.793-0.931) in the training cohort, an AUC of 0.826 (0.718-0.934) in the validation cohort, and an AUC of 0.826 (0.705-0.947) in the external validation cohort (**Table 3**; **Figures 4**, **5**).

**Figure 5:**
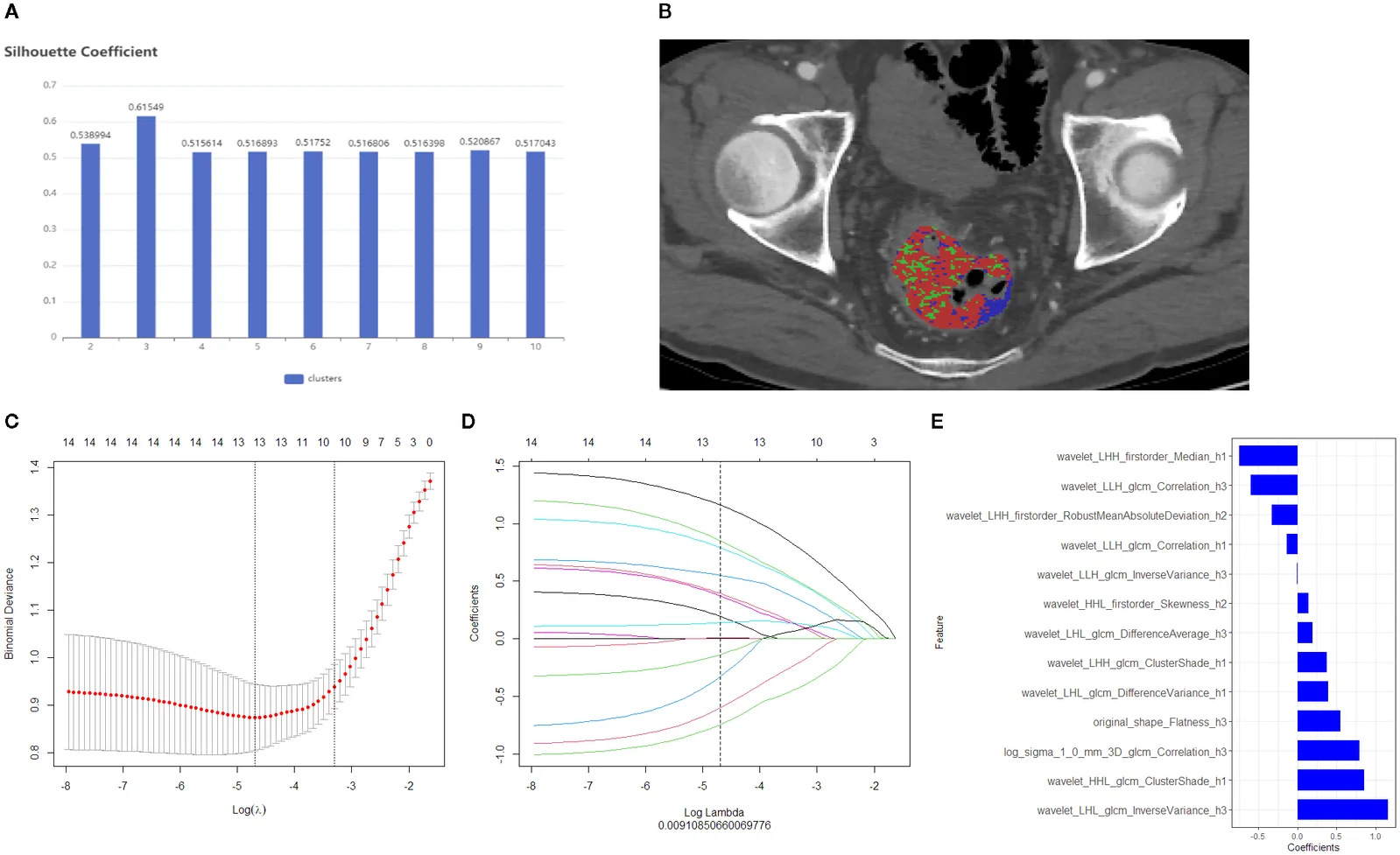
**(A)** Silhouette coefficient values corresponding to the number of clusters ranging from 2 to 10; **(B)** K-means clustering results with three clusters (K = 3); Feature dimensionality reduction and screening of the habitat radiomics model. **(C, D)** Schematic diagrams of feature screening and dimensionality reduction, **(E)** Features incorporated into the model after dimensionality reduction and screening as well as the weight of each feature).

### 3.4 Construction and validation of the combined model

Clinically significant clinical indicators including CEA, KRAS and habitat radiomics scores in the training set were incorporated into multivariate Logistic regression analysis. The results demonstrated that CEA (OR = 3.747, 95%CI: 1.332-10.619, P = 0.013), KRAS (OR = 3.015, 95%CI: 1.087-8.364, P = 0.034), and habitat radiomics score (OR = 2.094, 95%CI: 1.556-2.819, P<0.001) were independent risk factors, based on which the combined model was established. The AUC values of the combined model in the training cohort, internal validation cohort and external validation cohort were 0.883 (0.812-0.954), 0.846 (0.748-0.945) and 0.851 (0.752-0.951) respectively, which were numerically higher than those of clinical model, radiomics model and habitat model (**Table 3**; **Figure 4**). DeLong test revealed statistically significant differences between the combined model and the clinical model in the training, internal validation and external validation cohorts (P<0.05), whereas no statistically significant differences were observed between the combined model and the radiomics model as well as the habitat model (P>0.05). Therefore, a nomogram was plotted for the combined model (**Figure 6**). The calibration curves of both the training and internal validation cohorts exhibited favorable calibration performance (**Figure 7**). The Hosmer-Lemeshow test for goodness-of-fit of this model yielded non-significant statistical results (training set: χ^2^ = 1.019, P = 0.601; internal validation set: χ^2^ = 4.378, P = 0.112; external validation set: χ^2^ = 1.867, P = 0.393), indicating that the model had no deviation from perfect fitting. Decision curve analysis (DCA) curves illustrated that the combined model achieved higher clinical net benefit in predicting synchronous liver metastasis in patients with rectal cancer compared with the clinical model, radiomics model and habitat model (**Figure 8**).

**Figure 6:**
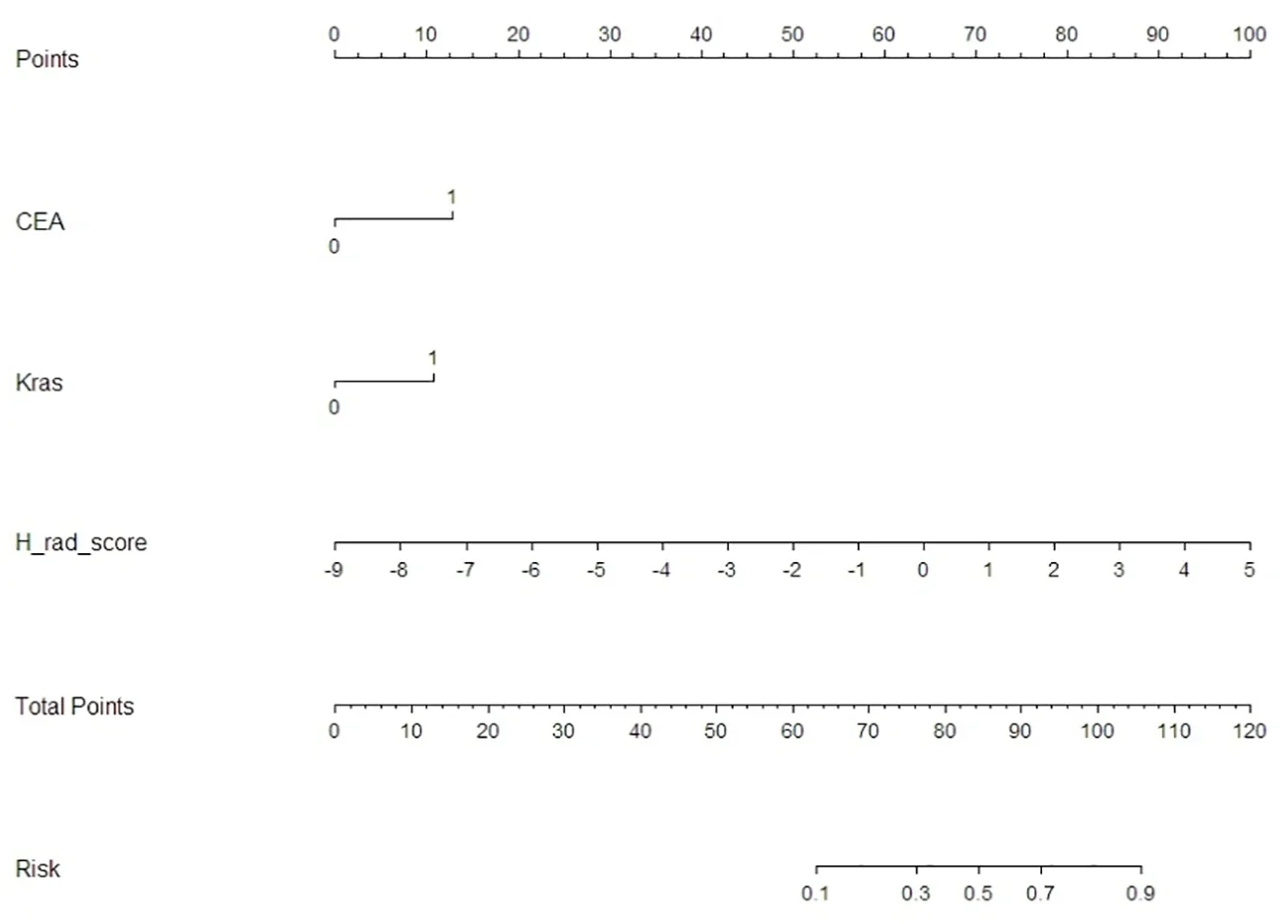
Nomogram of the combined model, H_rad_score: Habitat radiomics score.

**Figure 7:**
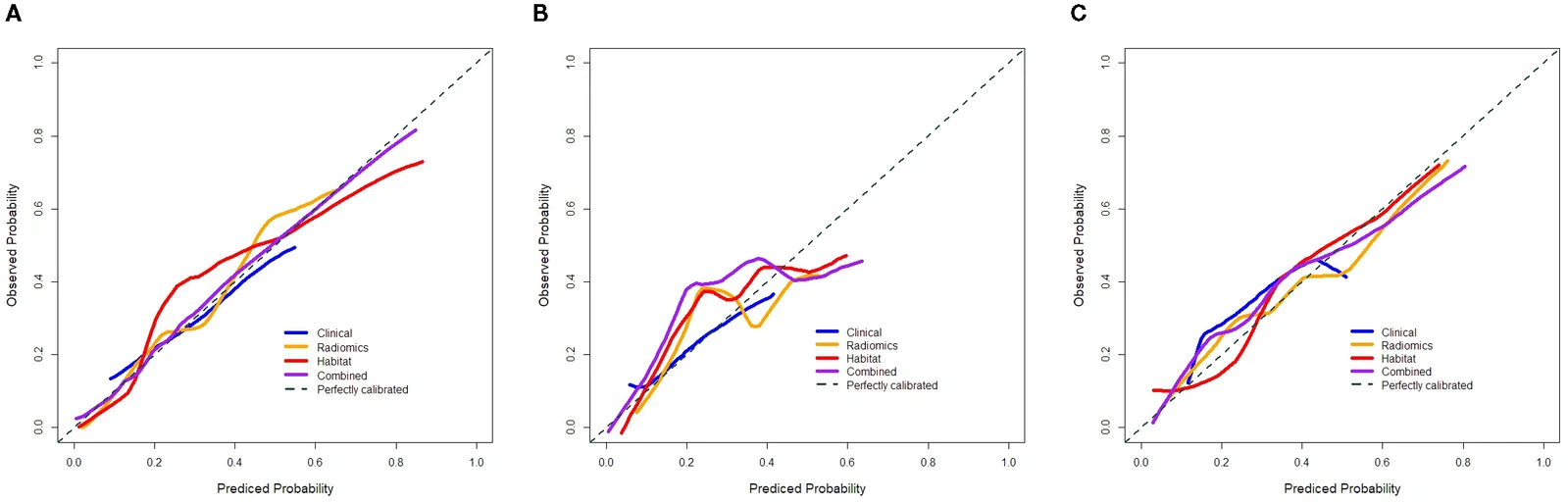
Calibration curves of each model for the training set **(A)**, validation set **(B)** and external validation set **(C)**.

**Figure 8:**
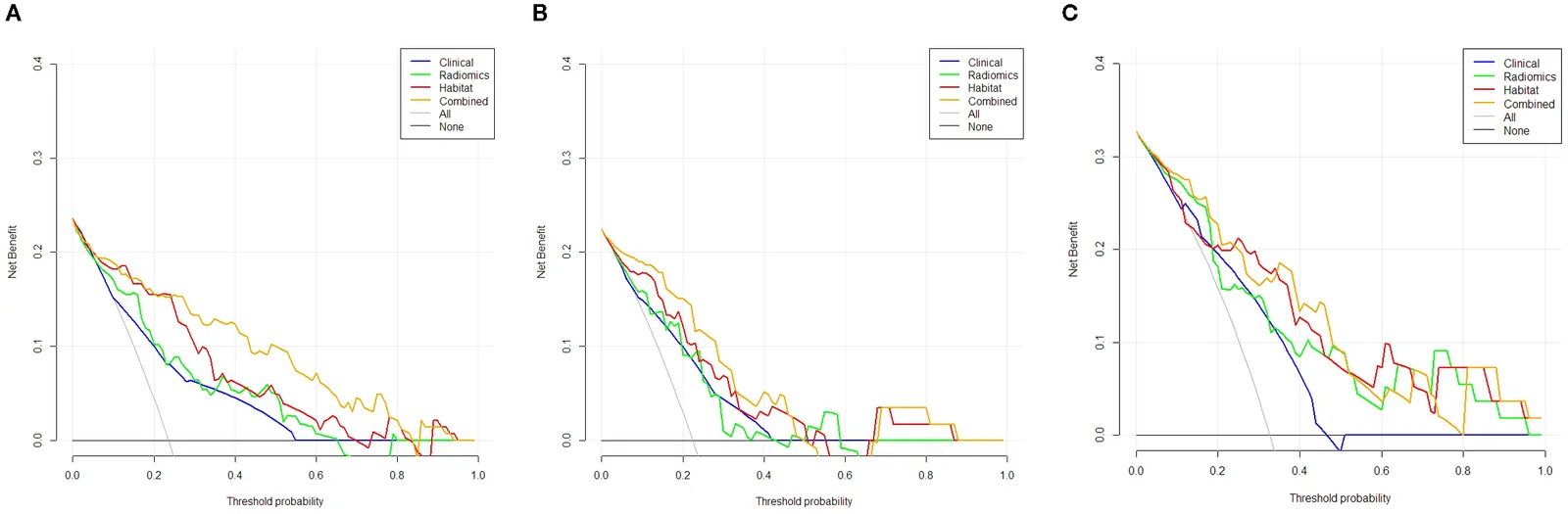
Decision curve analysis of each model in the training set **(A)**, validation set **(B)**, and external validation set **(C)**.

## 4 Discussion

This study explored the construction of a diagnostic model combining a CT radiomics habitat model with clinical indicators to distinguish between patients with RC with and without SLM. The diagnostic performance of the habitat model was numerically better than that of conventional radiomics models, while a combined model integrating independent clinical risk factors achieved even better diagnostic efficiency, with AUCs of 0.883, 0.846, and 0.851 in the training, validation, and external validation cohorts, respectively. In addition, we established and visualized a nomogram prediction model to provide an intuitive and accurate quantitative reference for clinicians to assess the risk of SLM in patients with RC. Notably, this was a dual-center study, which helped to enhance the generalizability of the model.

MRI has excellent soft tissue resolution and can accurately assess the local invasion depth of RC; however, it has high examination costs, as well as contraindications for scanning and substantial equipment-operating expenses. In contrast, CT is the routine imaging modality for screening SLM of RC, with the advantages of low equipment-maintenance costs and straightforward scanning procedures, making it highly suitable for use in primary medical institutions. Accordingly, this study constructed radiomics and habitat models based on CT images, to improve the applicability of this diagnostic protocol in primary clinical settings.

The current results identified the CEA index as an independent risk factor for predicting liver metastasis in patients with RC, consistent with previous studies (19–21). *KRAS* gene mutation acts as a biomarker for a poor response to anti-epidermal growth factor antibodies (22, 23), and numerous studies have also demonstrated a close association between *KRAS* and the prognosis of patients with RC, with *KRAS* mutations associated with a higher risk of SLM (24). The present study revealed that *KRAS* mutation was an independent risk factor for liver metastasis in patients with RC, signifying a higher tendency for SLM in patients with *KRAS* mutations, and suggesting that this gene may be linked to the invasiveness of RC.

This study was based on the analysis of CT images. The available conventional quantitative imaging indicators were relatively limited compared with previous studies, and the interpretation of traditional imaging indicators inevitably involves subjective bias. However, radiomics technology can mine high-throughput deep features from images, effectively making up for the insufficient data on conventional imaging indicators, minimizing errors caused by manual subjective interpretation, and improving the objectivity and accuracy of model evaluation. Liu et al. (20) successfully predicted SLM in patients with RC by extracting radiomics features from preoperative MRI-T2-weighted images; however, their combined model achieved an AUC value of 0.827 in the validation cohort, which was lower than the value in the present study. The reason for this difference is that traditional radiomics treats tumors as homogeneous masses and ignores intratumoral heterogeneity, while the current study used a habitat analysis method, which divided the tumors into distinct habitat subregions based on specific voxel values, enabling a more refined analysis of intratumoral heterogeneity. Mou et al. (25) predicted SLM in patients with RC via feature extraction from endorectal ultrasound images. However, the acquisition of ultrasound images depends on the experience of the examining physicians; different physicians’ observations and diagnosis of the same lesion are affected by their respective subjective experiences, leading to the biased extraction of imaging features. This imposes certain limitations on the stability and generalizability of relevant prediction models, making their wide application in clinical practice difficult.

Compared with conventional radiomics, habitat imaging emphasizes subregional analysis, which divides the entire tumor into distinct subregions according to different voxels and enables the more precise characterization of intratumoral heterogeneity associated with tumor progression and invasiveness (26). Tumor-habitat analysis can mine differential intratumoral heterogeneity information from multiple perspectives and effectively capture imaging biological signatures that are highly correlated with the patient’s prognostic status and clinical treatment response, with superior capacity for resolving tumor pathological features (27). Many previous studies have suggested that habitat analysis may offer clinical advantages. Zhang et al. (28) applied habitat analysis to the prediction of pathologic complete response in breast cancer and demonstrated that a habitat model had significantly better predictive performance than conventional intratumoral radiomics and peritumoral models. Ding et al. (29) conducted habitat analysis based on dynamic contrast-enhanced MRI to construct a preoperative prediction model for axillary lymph node metastasis in patients with breast cancer. They confirmed that the habitat model outperformed conventional radiomics models and enabled accurate, non-invasive assessment of preoperative lymph node metastasis risk in these patients. Chen et al. (30) developed a prediction model based on habitat analysis in 164 patients with intrahepatic cholangiocarcinoma. This model could accurately evaluate patients’ preoperative lymph node metastasis status and provide evidence for individualized diagnosis and treatment decisions, and had important clinical-guidance value for optimizing patients’ treatment regimens and improving their long-term prognosis. The habitat model established by Ao et al. (31) accurately predicted T2–T3 staging of RC, further expanding the application of habitat analysis for the precise stratified evaluation of RC. Numerous studies have thus confirmed (32–34) the superiority of habitat analysis compared with conventional radiomics for mining intratumoral heterogeneity information and for tumor evaluation and prediction, consistent with the conclusions of the present study. The current findings confirmed that the predictive efficacy of habitat models surpassed that of conventional radiomics models. However, research on habitat models for liver metastasis of RC remains relatively scarce. The results of this study can thus help to fill the gaps in existing research and provide a reference for improving the clinical diagnosis and treatment of liver metastasis in patients with RC. This study also integrated clinical indicators, including *CEA* and *KRAS*, with habitat radiomics scores to establish a combined prediction model and visualized the model via a nomogram, to provide an intuitive, convenient, and quantifiable assessment tool to help clinicians to evaluate the risk of liver metastasis of RC.

This study had some limitations. First, this was a retrospective study and data collection was based on previous case records, inevitably leading to selection bias, although sample size was pre-calculated for an expected AUC of 0.80, the final number of SLM-positive patients was relatively small. Second, the sample size was relatively small, and further large-sample prospective studies are needed to verify the generalizability and clinical applicability of the model. Third, the habitat radiomics model was constructed based on arterial-phase enhanced CT images, without incorporating multi-phase image information. Follow-up studies combining multi-phase imaging data are expected to further improve the diagnostic efficiency of the model. Finally, the case-collection period of the study was relatively long, and to avoid confounding problems such as non-standard recording of previous clinical data and missing data, relatively limited clinical indicators were included in the study. Further prospective studies are needed to standardize the clinical data-collection process, expand the feature dimensions, and provide data support for the construction of more-comprehensive and accurate prediction models.

In summary, this study constructed a novel predictive model by combining the clinical tumor markers *CEA* and *KRAS* with habitat radiomics scores, which could effectively and accurately assess the risk of SLM in patients with RC. The predictive performance of the habitat model was numerically better than that of the traditional radiomics model. Moreover, a combined model integrating clinical tumor indicators could further improve the diagnostic performance, with better predictive efficiency, providing new technical approaches and clinical strategies for the non-invasive and precise preoperative evaluation of SLM in patients with RC.

## Data Availability

The raw data supporting the conclusions of this article will be made available by the authors, without undue reservation.
