# Supplementary material for: A model integrating CEA and KRAS mutation statuses and computed tomography tumor-habitat radiomics predicts synchronous liver metastases in patients with rectal cancer: a dual-center study
Source: Front Oncol. 2026 Oct 28;16:1927131. doi: 10.3389/fonc.2026.1927131 (PMC13751435; doi:10.3389/fonc.2026.1927131)
Supplement: Supplementary file 1 [file DataSheet1.pdf]

Sample size: area under ROC curve

Options

Type I error (Alpha, Significance)	0.05
Type II error (Beta, 1-Power)	0.10

Data

Area under ROC curve	0.8
Null Hypothesis value	0.5
Ratio of sample sizes in negative / positive groups	3.24

Result

Number of positive cases required:	12
Number of negative cases required:	39
Total sample size (both groups together)	51

Table

		Type I Error - Alpha			
		0.20	0.10	0.05	0.01
Type II Error - Beta	0.20	5 + 17	7 + 23	9 + 30	14 + 46
	0.10	7 + 23	10 + 33	**12 + 39**	17 + 56
	0.05	9 + 30	12 + 39	14 + 46	20 + 65
	0.01	13 + 43	16 + 52	19 + 62	26 + 85
